# Supplementary material for: Exogenous Melatonin Alters Stomatal Regulation in Tomato Seedlings Subjected to Combined Heat and Drought Stress through Mechanisms Distinct from ABA Signaling
Source: Plants (Basel). 2023 Mar 3;12(5):1156. doi: 10.3390/plants12051156 (PMC10005520; doi:10.3390/plants12051156)
Supplement: Supplementary file 1 [file plants-12-01156-s001.zip › plants-2234500-supplementary.pdf]

Supplementary material

Table S1: Mean and SE of additional stomatal parameters. All measures of lenth and width are in units of µm. Measures of area are in units of μm^2

	Control				Melatonin			
	26°C irrigated	26°C drought	38°C irrigated	38°C drought	26°C irrigated	26°C drought	38°C irrigated	38°C drought
**Moderate stress (25 DAS)**								
Abaxial stomata length	30.1 ± 0.4	29.5 ± 0.4	29.7 ± 0.5	31.8 ± 0.5	29.1 ± 0.4	29.2 ± 0.4	31.6 ± 0.5	31 ± 0.4
Abaxial stomata width	17.5 ± 0.2	17 ± 0.2	19.5 ± 0.2	19.7 ± 0.2	17.8 ± 0.2	17.5 ± 0.2	20.5 ± 0.3	20.7 ± 0.2
Abaxial stomata size	531 ± 12	505 ± 11	578 ± 12	628 ± 13	521 ± 11	514 ± 11	652 ± 17	646 ± 14
Abaxial pore length	20.6 ± 0.4	20.2 ± 0.5	20.3 ± 0.4	21.4 ± 0.5	19.9 ± 0.4	19.4 ± 0.4	21.1 ± 0.5	20.8 ± 0.5
Abaxial pore width	3 ± 0.1	2.7 ± 0.1	5.5 ± 0.2	4 ± 0.2	3.4 ± 0.1	2.8 ± 0.1	5.1 ± 0.3	5.4 ± 0.2
Adaxial stomata length	31.7 ± 0.5	30.8 ± 0.4	32.2 ± 0.4	33.3 ± 0.6	31.3 ± 0.5	30.9 ± 0.4	35.9 ± 0.8	31.7 ± 0.5
Adaxial stomata width	16.2 ± 0.3	16.1 ± 0.2	17 ± 0.2	17.8 ± 0.3	16.4 ± 0.2	16 ± 0.3	17.5 ± 0.4	18.6 ± 0.3
Adaxial pore length	19.6 ± 0.5	18 ± 0.4	21.1 ± 0.3	21.8 ± 0.6	19.2 ± 0.4	17.1 ± 0.4	23.3 ± 0.9	20.7 ± 0.5
Adaxial pore width	1.8 ± 0.1	1.9 ± 0.1	3 ± 0.2	3 ± 0.2	2 ± 0.1	1.8 ± 0.1	2.9 ± 0.3	3.7 ± 0.2
**Severe stress (27 DAS)**								
Abaxial stomata length	30.6 ± 0.4	29.8 ± 0.4	33.7 ± 0.4	30.1 ± 0.4	30.1 ± 0.4	29.9 ± 0.4	33.9 ± 0.5	31.2 ± 0.4
Abaxial stomata width	17.6 ± 0.2	17 ± 0.2	22.4 ± 0.4	19 ± 0.2	17.8 ± 0.2	16.9 ± 0.2	21 ± 0.3	19.9 ± 0.3
Abaxial stomata size	543 ± 11	506 ± 10	756 ± 17	575 ± 13	535 ± 9	508 ± 11	716 ± 16	622 ± 14
Abaxial pore length	19.8 ± 0.4	19.8 ± 0.5	23.8 ± 0.5	20.5 ± 0.4	19.7 ± 0.4	20 ± 0.4	24.1 ± 0.5	22.5 ± 0.4
Abaxial pore width	2.7 ± 0.1	2.5 ± 0.1	5.1 ± 0.3	2.8 ± 0.2	3 ± 0.2	2.5 ± 0.1	4.5 ± 0.2	3 ± 0.2
Adaxial stomata length	32.5 ± 0.6	32.9 ± 0.5	34.3 ± 0.4	30.3 ± 0.4	30.8 ± 0.5	32.4 ± 0.6	36 ± 0.5	33.2 ± 0.5
Adaxial stomata width	17 ± 0.3	16.6 ± 0.3	20.2 ± 0.3	17 ± 0.2	16.4 ± 0.2	16.1 ± 0.2	19.7 ± 0.3	18.7 ± 0.3
Adaxial pore length	19.5 ± 0.6	19.7 ± 0.5	23 ± 0.3	20 ± 0.4	18.1 ± 0.5	19.1 ± 0.6	24.8 ± 0.5	21.8 ± 0.3
Adaxial pore width	2.6 ± 0.2	2.3 ± 0.1	4.3 ± 0.3	2.1 ± 0.1	2.2 ± 0.1	1.7 ± 0.1	2.9 ± 0.2	2.6 ± 0.2

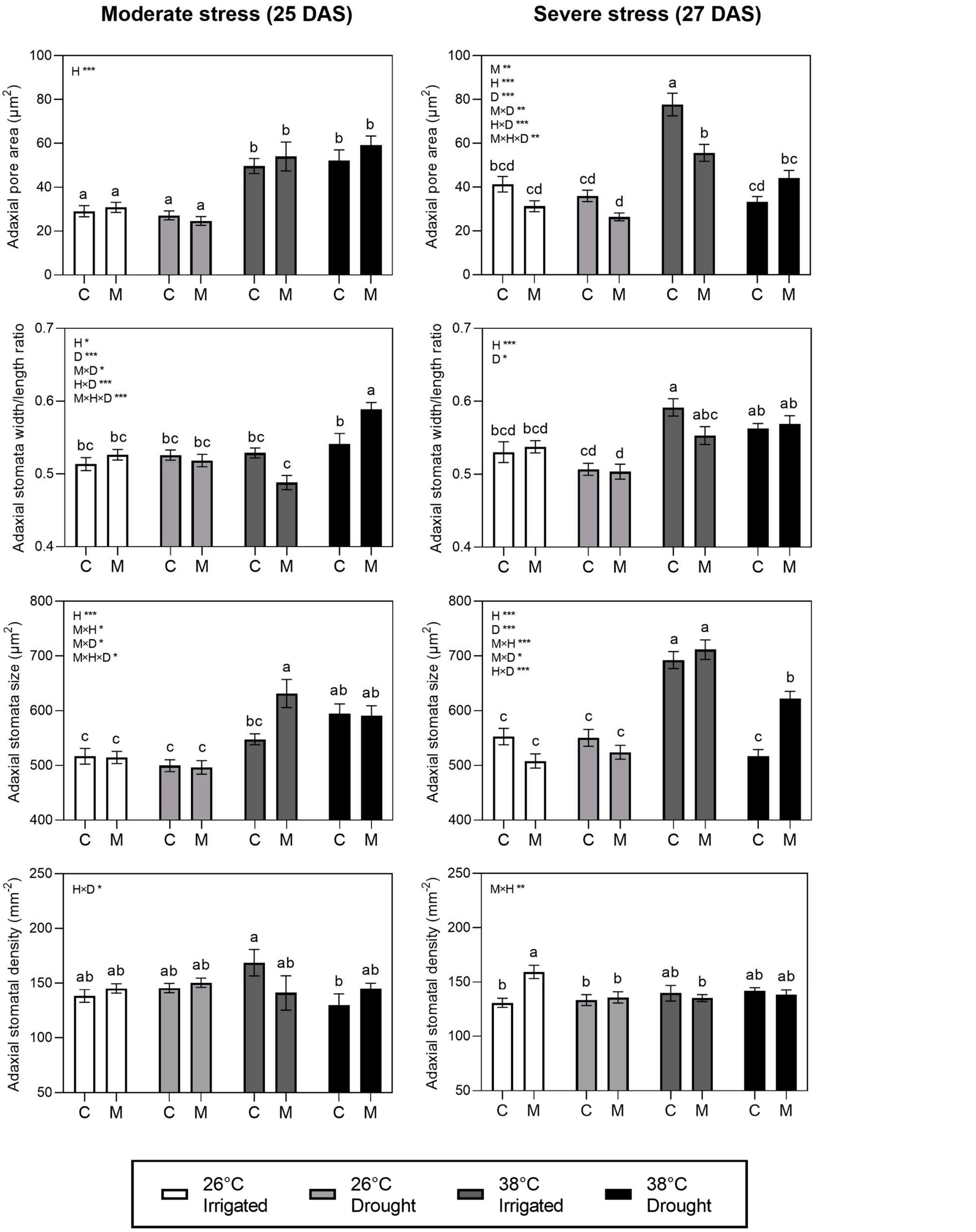

Figure S1. Stomatal anatomy from the adaxial leaf side on plants during moderate stress (drought stressed at SRWC ~50% and heat stress for 1 day) and severe stress (drought stressed at SRWC ~20% and heat stress for 3 days). Values are mean +/− SE. Letters indicates differences between groups within each stress level significant at P < 0.05 in the post hoc multiple comparisons.

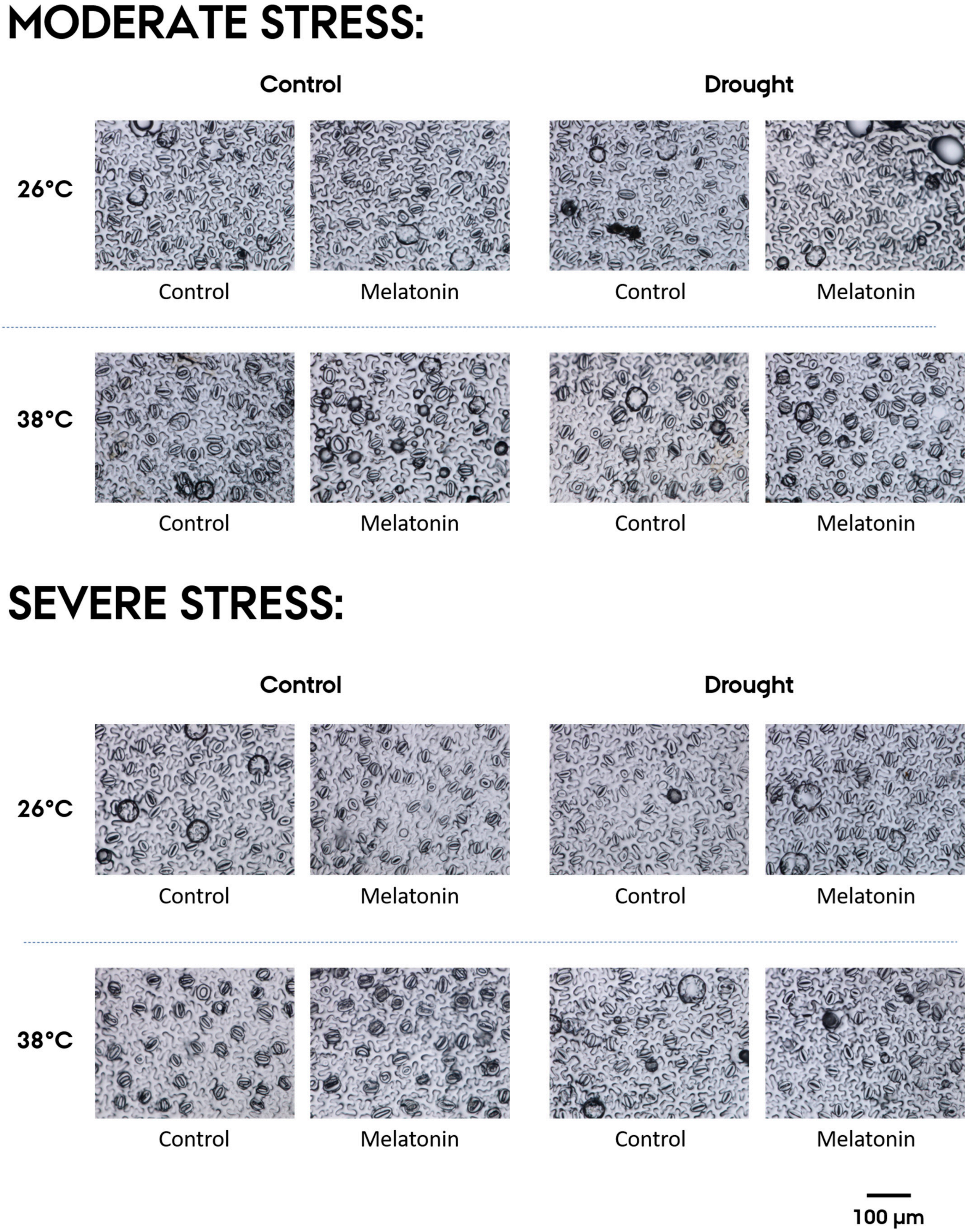

Figure S2. Uncropped microscope images for illustrating stomatal density

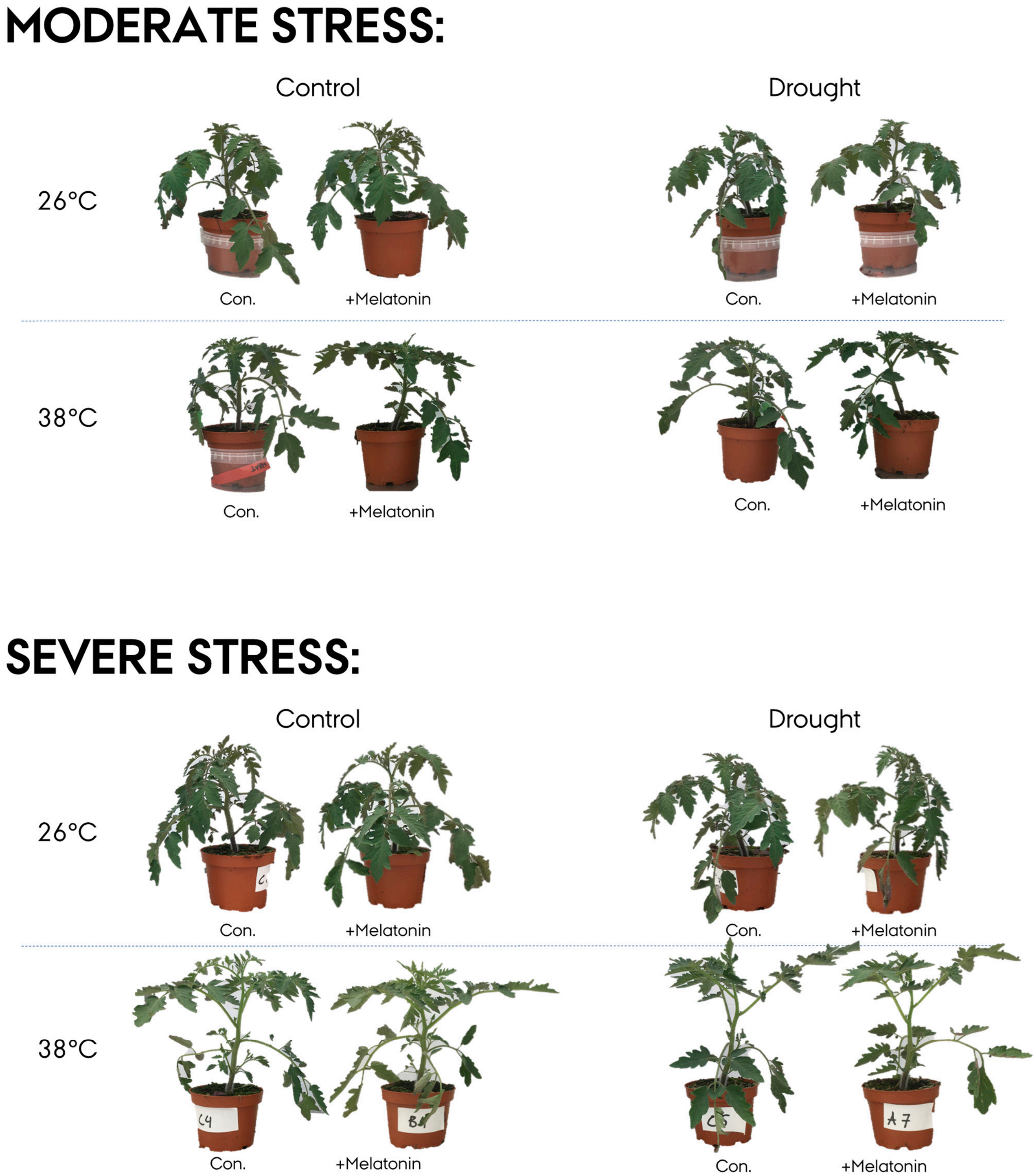

Figure S3. Plants photographed at the time of measuring/sampling (25 and 27 DAS)
